# The Influence of Cell Type and Culture Medium on Determining Cancer Selectivity of Cold Atmospheric Plasma Treatment

**DOI:** 10.3390/cancers11091287

**Published:** 2019-09-01

**Authors:** Eline Biscop, Abraham Lin, Wilma Van Boxem, Jinthe Van Loenhout, Joey De Backer, Christophe Deben, Sylvia Dewilde, Evelien Smits, Annemie Bogaerts

**Affiliations:** 1 PLASMANT Research Group, Department of Chemistry, University of Antwerp, 2610 Antwerp, Belgium; 2 Center for Oncological Research, University of Antwerp, 2610 Antwerp, Belgium; 3 Department of Biomedical Sciences, University of Antwerp, 2610 Antwerp, Belgium

**Keywords:** cold atmospheric plasma, selectivity, plasma-treated liquid, dielectric barrier discharge

## Abstract

Increasing the selectivity of cancer treatments is attractive, as it has the potential to reduce side-effects of therapy. Cold atmospheric plasma (CAP) is a novel cancer treatment that disrupts the intracellular oxidative balance. Several reports claim CAP treatment to be selective, but retrospective analysis of these studies revealed discrepancies in several biological factors and culturing methods. Before CAP can be conclusively stated as a selective cancer treatment, the importance of these factors must be investigated. In this study, we evaluated the influence of the cell type, cancer type, and cell culture medium on direct and indirect CAP treatment. Comparison of cancerous cells with their non-cancerous counterparts was performed under standardized conditions to determine selectivity of treatment. Analysis of seven human cell lines (cancerous: A549, U87, A375, and Malme-3M; non-cancerous: BEAS-2B, HA, and HEMa) and five different cell culture media (DMEM, RPMI1640, AM, BEGM, and DCBM) revealed that the tested parameters strongly influence indirect CAP treatment, while direct treatment was less affected. Taken together, the results of our study demonstrate that cell type, cancer type, and culturing medium must be taken into account before selectivity of CAP treatment can be claimed and overlooking these parameters can easily result in inaccurate conclusions of selectivity.

## 1. Introduction

Chemotherapy and radiotherapy are two major pillars in the management of cancer. Significant efforts to make these treatments more selective are ongoing, with the intention of reducing side-effects of therapy [1]. Despite the remarkable evolution of conventional cancer therapies, they are still met with limitations as evidenced by the fact that cancer remains the second leading cause of death worldwide [2]. As a result, new alternative or additional cancer treatment methods are also under investigation to support current treatment strategies.

Cold atmospheric plasma (CAP) has been investigated as novel cancer treatment strategy, and interest in the use of CAP for cancer treatment has been growing [3]. CAP is an ionized gas near room temperature, composed of various molecules, radicals, ions, electrons, and excited species [4]. Over the past decade, the anti-cancer capacity of CAP has been reported for multiple cancer types in vitro [5,6,7,8,9,10,11], while in animal models, CAP treatment has reduced tumor burden in mice and increased survival [12,13]. Nowadays, several CAP devices are being used in the clinic for treatment of cancerous lesions [14,15,16].

The current understanding of CAP mechanisms for effecting biological response is that the reactive oxygen and nitrogen species (RONS) generated by CAP elicit oxidative damage to the cell, resulting in cell death [17,18]. According to this understanding, CAP treatment has been hypothesized to be selective for cancer, as the disturbance of the oxidative balance occurs more easily in cancer cells compared to healthy cells [19,20]. Additionally, cancer cells have more aquaporins and less cholesterol in their cellular membrane, which contributes to the diffusion of certain CAP-generated RONS through the membrane and facilitates pore formation, respectively [21,22,23,24]. Furthermore, Bauer and Graves proposed a theory where the initial concentration of singlet oxygen, produced by the plasma, triggers cells to generate higher concentrations of secondary singlet oxygen, which leads to the inactivation of catalase in the cell membrane [25]. The inactivation of catalase can also play an important role in the selectivity, as this allows reactivation of intercellular ROS/RNS-dependent, apoptosis-inducing signaling within the population of tumor cells [25]. To date, however, the underlying mechanisms of CAP selectivity are not yet fully understood, and furthermore, the selectivity has not been fully validated.

While several papers claim that CAP selectively kills cancer cells in vitro, retrospective analysis of these papers reveals that definitive proof is rather scarce. This is largely due to the discrepancies between treatment conditions for cancerous and non-cancerous cells. In several cases, the cell culture media used for cancerous and non-cancerous cells were not the same, while in other studies, the cell culture media was not specified at all [8,26,27,28,29,30]. It is understandable that non-cancerous cells normally require more advanced cell culture media with additional organic compounds compared to cancer cells, but the different media have disparate buffering and antioxidant capacities [31]. In fact, the stability of RONS in different liquids has been thoroughly investigated [32,33] and Yan et. al showed that the presence of cysteine and methionine can significantly degrade CAP-generated RONS [31]. Since the working mechanism of CAP involves disrupting the oxidative balance of cells via RONS generation, changes in media composition could impede their production and delivery, subsequently affecting biological outcome. Therefore, the observed selectivity of CAP treatment could actually result from variation in media and not from intrinsic sensitivity of cancerous and normal cells. In other studies, the selectivity of CAP treatment was claimed, but comparisons were made with different cell types (e.g., epithelial cancer cells with non-cancerous fibroblast cells) and even tissue types (e.g., comparison of ovarian cancer cells with non-cancerous lung cells) [27,30,34,35,36]. Due to the different physiological characteristics of distinct tissues [37], comparisons between equivalent cell types must also be made before selectivity of treatment can be claimed [37].

Taken together, in order to avoid false claims of selectivity for CAP treatment, the potentially confounding factors found in previous work, must be investigated. Therefore, our goals in this study were to address the following: What are the influences of the cell and cancer types on selectivity experiments? What are the influences of cell culture medium on selectivity experiments? Finally, when the proper comparisons are made, is CAP treatment more selective against cancerous cells compared to their normal, non-cancerous counterparts? In this study, two well-established methods of CAP treatment were studied—‘direct’ and ‘indirect’ treatment [38]. In the direct case, CAP was generated directly onto cells, while in the indirect case, a liquid (e.g., saline) was enriched with RONS following CAP treatment, and this plasma-treated liquid (PTL) was then delivered to cells or tissue. The selectivity of both treatment methods for three different cancer types (lung cancer, skin cancer, and brain cancer) was analyzed by comparing their survival 24 hours after treatment with that of their non-cancerous counterparts. Next, the cytotoxic effects of CAP treatment on cells using five different cell culture media—i.e., two ‘standard’ cell culture media and three ‘more advanced’ cell culture media for cancerous and non-cancerous cells, respectively, were analyzed. Our results showed that both the cell type and cancer type, as well as the cell culture medium, can have a substantial influence on the outcome of experiments. When analyzing selectivity of CAP treatment in the correct way (with cancerous and non-cancerous cells from the same tissue, the same cell type, and cultured in the same medium), appreciable selectivity was not observed in this study.

## 2. Results

### 2.1. Influence of Cell Type and Cancer Type on Cell Viability

**Figure 1:**
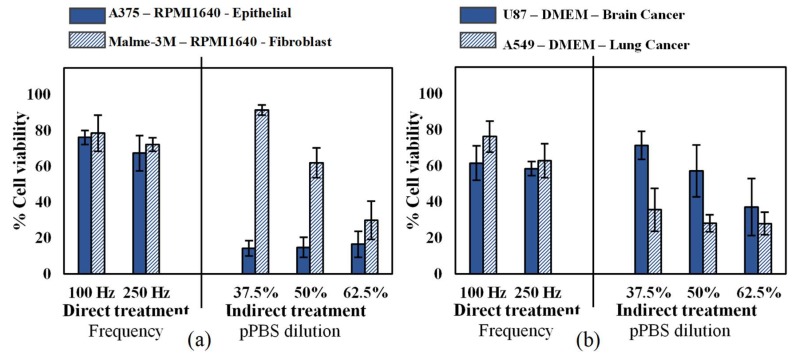
Analysis of the influence of the cell type and cancer type on both direct treatment (FE-DBD at two different frequencies) and indirect treatment (pPBS in three different dilutions). (**a**) Comparison of an epithelial cell line (A375) with a fibroblast cell line (Malme-3M), which are both skin cancer cell lines. (**b**) Comparison of a brain cancer cell line (U87) with a lung cancer cell line (A549). For both figures, the cells were cultured in the same cell culture medium and treated with exactly the same conditions. Therefore, the only variables tested were (**a**) cell type and (**b**) cancer type. Data are represented as mean ± standard deviation (SD) of three independent experiments with at least two replicates. Statistical significance of all treatment conditions was compared to untreated. *p < 0.05, **p < 0.01, and ***p < 0.005 (one-way ANOVA).

Since different cell types have different responses to oxidative stress [39], we first investigated the cytotoxic effect of CAP on two human malignant melanoma cell types: an epithelial cell line (A375, derived from skin) and a fibroblast cell line (Malme-3M, derived from a metastatic site on the lung), according to the American Type Culture Collection (ATCC). As these cells were cultured in the same medium and under the same conditions, the cell type was the only variable in this experiment. Our results showed that while there was no significant difference in sensitivity for the cell lines with direct CAP treatment, the epithelial cells were more sensitive to indirect treatment compared to malignant fibroblasts (Figure 1a). To further evaluate the cytotoxic effects of CAP on different cancer types, we treated two additional human epithelial cells, U87 glioblastoma and A549 lung carcinoma. All cancer types were equally sensitive to direct CAP treatment, but the U87 was less sensitive to indirect treatment (Figure 1b). Therefore, it is clear that cell sensitivity to indirect CAP treatment is influenced by both cell type and cancer type, while this impact seems not present with direct treatment.

### 2.2. Influence of Cell Culture Media on Cell Viability

To determine the importance and influence of cell culture medium when assessing selectivity of treatment, we evaluated the cytotoxicity of both direct and indirect treatment for the A549 and A375 cell lines, in five different media. In order to ensure that the different media alone did not significantly affect cell growth and death, the cytotoxicity assay was performed on cells 24 hours after incubation and cell density in the different media was compared to that of their recommended medium: DMEM for A549 and RPMI for A375 (Figure S1, Supplementary Information). The A375 cells were able to grow in all media with a similar growth rate to that in RPMI1640. However, there was a statistically significant decrease in cell growth of the A549 cells in BEGM compared to DMEM. This suggests that even without CAP treatment, certain cell processes are strongly influenced by the components of the cell culture medium. Due to this discrepancy on cell growth, selectivity of treatment cannot be determined for cases where normal, non-cancerous cells are grown in the BEGM medium. This was further validated when A549 and their normal counterparts (BEAS-2B) were cultured in the BEGM medium and treated with direct and indirect CAP (Figure S2, Supplementary Information). Therefore, this medium was removed from all subsequent experiments. For the other three media, the difference in growth rate was not significant (*p* > 0.05, details in Supplementary Information).

**Figure 2:**
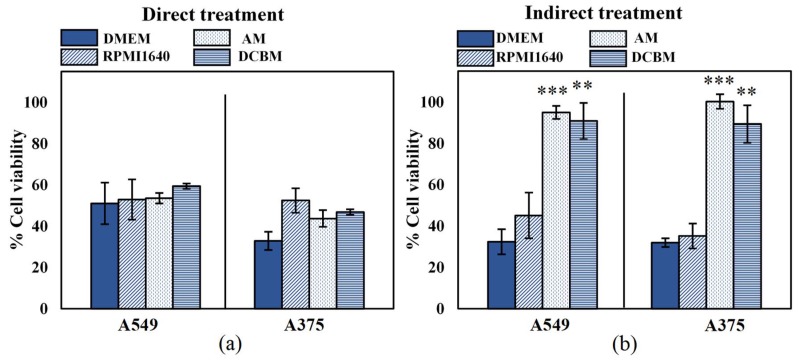
Influence of the cell culture medium on the direct and indirect plasma treatment of two cancer cell lines. (**a**) The direct plasma treatment was performed for 10 s, with a frequency of 500 Hz and a gap of 1 mm. (**b**) The indirect treatment was performed for 7 min treatment, with a gas flow rate of 3 slm and a gap of 10 mm. Data are represented as mean ± standard deviation (SD) of three independent experiments with at least two replicates. Statistical significance of all treatment conditions was compared to untreated. *p < 0.05, **p < 0.01, and ***p < 0.005 (one-way ANOVA).

The effect of direct CAP treatment was unaffected by the cell culture medium (Figure 2a), as the cell culture medium was removed during treatment. These results further indicated that the effect of direct CAP treatment was initiated during treatment and unaffected by the scavenging effects of the cell culture media added immediately afterwards. For the indirect treatment, cytotoxicity was significantly influenced by the cell culture media (Figure 2b). Cancer cells treated in the standard media (DMEM and RPMI1640) resulted in ≥50% cytotoxicity, but were unaffected when treated in advanced media used to culture normal, non-cancerous cells (AM and DCBM).

### 2.3. Influence of Cell Culture Media on Selectivity Evaluation of Indirect CAP Treatment

**Figure 3:**
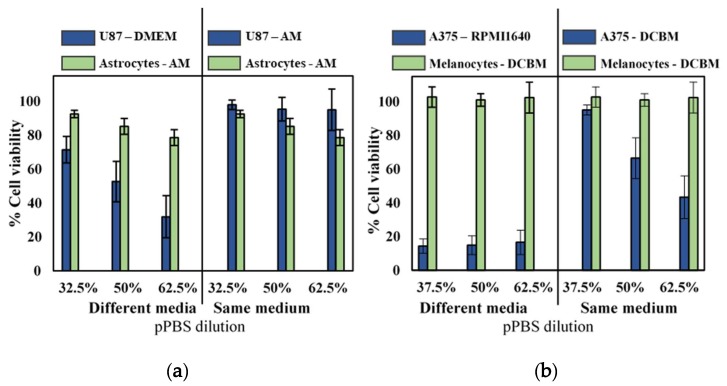
Analysis of the selectivity with the indirect treatment for brain cancer and skin cancer. A 5-min treatment with a gas flow rate of 1 slm and a gap of 6 mm was used to create the pPBS, which we further diluted. Comparison of (**a**) brain and (**b**) skin cancer cells in their common medium (blue solid bars on the left side) with non-cancerous cells in their common medium (green solid bar on the left side) appeared to show selectivity in all cases. However, when compared to the cancer cells in the advanced media (on the right side of the graph), the selectivity was not found. Hence, this clearly shows that the selectivity was affected by the cell culture medium. This is important to realize, to avoid drawing false conclusions. Data are represented as mean ± standard deviation (SD) of three independent experiments with at least two replicates. Statistical significance of all treatment conditions was compared to untreated. *p < 0.05, **p < 0.01, and ***p < 0.005 (one-way ANOVA).

To further validate selectivity of indirect CAP treatment and the influence of cell culture media, we compared cytotoxicity for the cancerous cell lines with their non-cancerous, complimentary cell lines (astrocytes and melanocytes for glioblastoma and melanoma, respectively) in both standard and advanced media. Experiments were performed with cells seeded in their recommended medium and with cells seeded in the same medium. As non-cancerous cells were incapable of being cultured in standard media, cancerous cells were grown in the more advanced media of their non-cancerous counterparts. When cultured and treated in their recommended media (different media), as commonly done in literature, it would appear that pPBS treatment resulted in significant selectivity (Figure 3). However, when both cell lines were cultured in the same media, selectivity was diminished. Only the A375 cell line showed cytotoxic effect in the more advanced media, but this was also reduced compared to treatment in standard media.

### 2.4. Influence of Cell Culture Media on Selectivity Evaluation of Direct CAP Treatment

Following previous Section 2.1, Section 2.2 and Section 2.3, it is clear that cell type, cancer type, and culture media are critical parameters for indirect CAP treatment. These parameters were also standardized for direct CAP treatment to evaluate their effect on selectivity.

**Figure 4:**
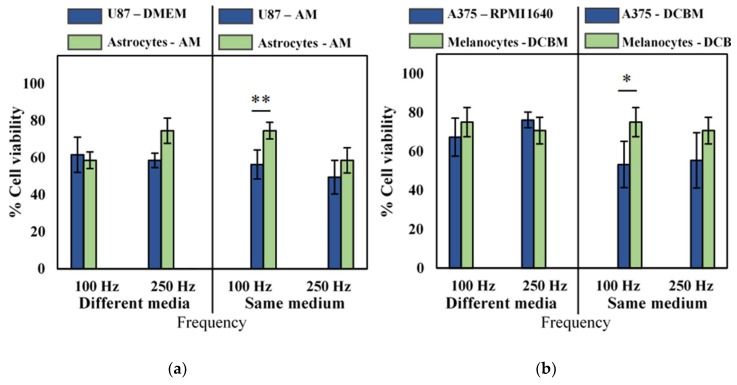
Analysis of selectivity with the direct treatment for brain cancer and skin cancer. A 10 second treatment with a gap of 1 mm and a frequency of either 100 Hz or 250 Hz was used to treat (**a**) brain and (**b**) skin cancer and non-cancerous cells. Data are represented as mean ± standard deviation (SD) of three independent experiments with at least two replicates. Statistical significance of all treatment conditions was compared to untreated. *p < 0.05, **p < 0.01, and ***p < 0.005 (one-way ANOVA).

Though we observed that the influence of different cell culture medium was not pronounced for direct CAP treatment, as described in Section 2.2, to be correct, we still performed our selectivity analysis of cancerous versus non-cancerous cell lines in both the same and different culture media. No selectivity was observed when cancerous and normal cells were cultured in their own recommended medium (Figure 4, different media), but when cancer cells were cultured and treated in the medium used for their non-cancerous counterparts, slight selectivity was observed in the U87 glioblastoma cell line, and the A375 melanoma cell line at lower intensity CAP treatment (Figure 4, same medium). However, at higher intensity treatment (250 Hz), no such selectivity was observed, as the difference was within error. Interestingly, our results suggest that there is an optimal regime for direct CAP treatment where selectivity can be achieved, above which the oxidative burden becomes too overwhelming for even normal cells to manage.

## 3. Discussion

Selectivity of CAP treatment for cancer is an important topic of research, but it has often been misconcluded. Multiple research groups claim to have found treatment conditions which selectively kill cancerous cells and leave non-cancerous cells unharmed. When examining those articles in more detail, we found critical discrepancies between the treatment conditions and origins of the cancerous and non-cancerous cells [8,26,27,28,29,30,34,35]. To ensure that these comparisons are not confounded by the discrepancies we identified, we analyzed their influence on cell viability after CAP treatment.

An important parameter often neglected in past studies, is the difference in cell type or cancer type [27,30,34,35]. For example, in one article, the authors compared ovarian cancer cells with non-cancerous lung cells [35]. In comparing the responses of two cell lines for both ovarian adenocarcinoma and non-cancerous lung tissue, they concluded that the cancer cell lines were more sensitive to the treatment than the non-cancerous cell line. However, Giordano et al. has reported a difference in gene expression profiles between lung cancer and ovarian cancer [40], which can result in differential responses to CAP treatment. Furthermore, the cell type of the cancerous cell lines and non-cancerous cell lines was different. The two ovarian cancerous cell lines used in that study, SKOV-3 and HRA, were epithelial cell lines, while the two non-cancerous lung cell lines, WI-38 and MRC-5, were fibroblasts. Epithelial cells and fibroblasts show different gene expression levels and can therefore give a different response to CAP treatment [41]. To analyze the influence of these parameters, we examined the difference in cell type and cancer type by comparing an epithelial and fibroblast cell line from the same cancer type and by comparing two epithelial cell lines from different cancer types. According to our results, both cell type and cancer type had an effect on sensitivity to CAP treatment. In light of these findings, it is clear that for analyzing selectivity, cells of the same cancer type and cell type should be chosen, and discrepancies between these two biological parameters could lead to misdirected conclusions of selectivity.

The influence of the cell culture medium is another important parameter often overlooked when determining the selectivity of CAP treatment. Therefore, the influence hereof on cell viability after CAP treatment was also investigated. The effect of two commonly used cell culture media (DMEM and RPMI1640) was compared with two more advanced cell culture media required for culturing non-cancerous cells (AM and DCBM). Our results showed that, when cells were cultured in the more advanced media, indirect CAP treatment was ineffective. This was likely in part due to the presence of more organic components in the advanced media, as non-cancerous cells require more nutrients and are much harder to grow in vitro [39]. RONS produced by CAP can react with these organic components before reaching the cells [31]. One component commonly added to most advanced media is sodium pyruvate, a known H_2_O_2_-scavenger [42,43]. For DMEM and RPMI1640, we ensured that no sodium pyruvate was present, but for the other two cell culture media, the composition was not specified by the manufacturer [44,45,46]. This would also explain why the cell culture media did not significantly influence cell viability following direct CAP treatment, as it was removed prior to treatment. These results highlight the influence of cell culture media on downstream biological effects following indirect CAP treatment. Taken together, it is clear that selectivity of CAP treatment cannot be evaluated for indirect plasma treatment when the cancer cells are cultured in medium different to that of non-cancerous cells.

Several papers claiming that CAP selectively kills cancerous cells cultured their cells in different media [8,26,27,28,29,30]. For example, one study cultured the A549 cells in DMEM, while their normal BEAS-2B cells were cultured in BEGM [30]. Since the authors saw more response to the treatment in their A549 cell line compared to the BEAS-2B cell line, they stated that their indirect CAP treatment was more selective. However, as evident from our results above, the sensitivity of A549 cells to indirect CAP treatment was reduced when cultured in BEGM compared to DMEM (Figure S2, Supplementary Information). Therefore, the reported selectivity was not cell line specific, but due to the different medium used to culture the cells. This strongly highlights the fact that cell culture medium plays an important role in indirect CAP treatment and must be standardized before claims of selectivity can be made.

Since the influence of the cell culture medium was less important for direct CAP treatment, we used this treatment method to analyze the selectivity of treatment for two cancer types: melanoma and glioblastoma. Interestingly, we observed that direct treatment preferentially affected cancer cells at lower intensity treatments (Figure 4), suggesting that selectivity depends on optimizing CAP treatment conditions that exploit the differences between normal and cancerous tissue. It is widely known that cancer cells have a higher proliferation rate, compared to non-cancerous cells. Healthy cells primarily produce energy through mitochondrial oxidative phosphorylation, while cancer cells predominantly produce their energy through a high rate of glycolysis followed by lactic acid fermentation, which benefits this high proliferation rate [47,48]. To sustain this fast growth rate, cancer cells require a ‘hyper metabolism’, which results in a higher level of basal intracellular ROS [49,50]. Simultaneously, cancer cells also maintain a high level of antioxidant activity, mainly reduced nicotinamide adenine dinucleotide phosphate (NADPH) and glutathione (GSH), to prevent build-up of ROS [47,49]. However, once the levels of ROS become excessively high through the addition of extra ROS, detrimental oxidative stress can occur, leading to cell death [51,52]. While randomized control clinical trials using pro-oxidant therapy are still ongoing, increasing evidence suggests that raising ROS levels through small molecules can selectively induce cancer cell death by disabling antioxidants [50,53,54]. Taken together with our observations, this would mean that the aim to reach selectivity of CAP treatment lies in the optimization of parameters and conditions to produce sufficient ROS to overwhelm the oxidative threshold in cancer cells, without reaching this threshold in the healthy cells.

It must be noted here that selectivity of CAP treatment may also depend on the RONS generated and delivered to the biological target. This is particularly important as direct and indirect CAP treatments generate a different cocktail of reactive species. With the indirect CAP treatment, a liquid is treated with CAP and then transferred to cells or tissue. Due to the time delay between treatment and application, only the long-lived species (mostly H_2_O_2_, NO_2_^−^, NO_3_^−^) remain in the liquid and reach the cells [36]. In the case of direct CAP treatment, the liquid is removed before treatment in order for CAP to be generated directly onto the cells, thereby enabling both the long-lived and short-lived (•OH, ^1^O_2_, O, O_3_, •NO, ONOO^−^) species to interact with the biological target [55]. Several reports have already demonstrated the importance of these short-lived species in direct CAP treatment for effecting cell death [55,56,57], though further fundamental investigations are still required, including the type of cell death modalities elicited.

The experiments performed in this study used cancer cell lines in 2D cultures. Cancer cell lines are derived from primary patient material and have provided important knowledge for cancer research. However, care must be taken when interpreting the results, as cell lines are genetically manipulated and therefore do not always accurately reflect the responses of primary cells [58]. Furthermore, comparison between two cell lines often involves comparison between two patient sources. To further investigate the selectivity in a more realistic manner, the cancer and healthy cells should be derived from the same patient [58]. Hasse et al. analyzed CAP treatment on cancer and healthy human tissue samples from head and neck cancer patients [58]. They found that CAP treatment of tumor tissue induced more apoptotic cells than in healthy tissue. This was accompanied by elevated extracellular cytochrome c levels in the tumor tissue [59]. Though this is probably the most representative in vitro model, human tissue samples cannot be preserved long-term and are therefore much more difficult to work with [60]. Another recently developed in vitro model is organoids [61,62]. These are 3D self-organizing organotypic structures, grown from tissue-derived adult stem cells. Organoids can be expanded long-term without losing their genetic and phenotypical stability [61,62]. Such 3D cell culture systems feature increased complexity for increased faithfulness to the in vivo environment and are above all fairly easy to work with [60]. When comparing these 3D organoids from healthy and cancerous tissue from the same patient, more representative results concerning the selectivity can be obtained compared to those obtained with 2D models.

The results from Hasse et al. are encouraging as it suggests that CAP treatment may indeed be selective when operated at certain regimes. As more studies using primary patient tissue and the proper 3D models start to emerge, the selectivity capacity of CAP treatment will become more clear. However, as it stands, it is evident from this study, several biological factors must be standardized and the proper comparisons must be made before these conclusions can be made.

## 4. Materials and Methods

### 4.1. Cell Culture and Plating

To evaluate the selectivity of the CAP treatment, we used three non-cancerous human cell lines as a model for healthy tissue (BEAS-2B—lung epithelial cell line, ATCC, Virginia; HA—human astrocytes, Sciencell, California; and HEMa—human epidermal melanocytes, ATCC, Virginia) and four cancer human cell lines (A549—Non-Small-Cell Lung Cancer cell line, ATCC, Virginia; U87—glioblastoma cell line, ATCC, Virginia; and A375, Malme-3M—melanoma cell lines, ATCC, Virginia). A549 and U87 were cultured in Dulbecco’s modified Eagle medium (DMEM) (Life Technologies, California, 10938025), A375 and Malme-3M were cultured in Roswell Park Memoriam Institute 1640 (RPMI1640) (Life Technologies, 52400025), BEAS-2B was cultured in Bronchial Epithelial Growth Medium (BEGM) (Lonza, Basel, CC-3170), the astrocytes were cultured in Astrocyte Medium (AM) (Sciencell, California, 1801) and the melanocytes were cultured in Dermal Cell Basal Medium DCBM (ATCC®, Virginia, PCS-200030TM). DMEM and RPMI1640 were supplemented with 10% Fetal Bovine Serum (FBS) (Gibco^TM^ FBS, Life Technologies, 10270098), 2 mM L-glutamine (Gibco^TM^, Life Technologies, 25030081), 100 units/mL penicillin, and 100 µg/mL streptomycin (Life Technologies, 15140163). According to the manufacturer’s protocol, BEGM was supplemented with 2 mL Bovine Pituitary Extract (BPE), 0.50 mL insulin, 0.50 mL hydrocortisone, 0.50 mL GA-1000, 0.50 mL retinoic acid, 0.50 mL transferrin, 0.50 mL triiodothyronine, 0.50 mL epinephrine, and 0.50 mL human epidermal growth factor (hEGF) (Lonza, CC-4175), AM was supplemented with 2% fetal bovine serum (Sciencell, 0010), 5 mL astrocyte growth supplement (Sciencell, 1852), and 5 mL of a penicillin/streptomycin solution (Sciencell, 0503). Finally, DCBM was supplemented with 5 µg/mL recombinant human insulin, 50 µg/mL ascorbic acid, 1 µM epinephrine, 1.5 mM calcium chloride, 1 mL peptide growth factor, and 5 mL M8 supplement (ATCC^®^, PCS-200042TM). The cells were incubated at 37 °C in a 5% CO2 humidified atmosphere. All media were prepared according to the recommendation for each cell line [44,45,46].

For the indirect treatment, the cells were seeded in 96-well plates with a density of 2500 cells per well for A549, BEAS-2B, and A375; 3000 cells per well for U87; and 6000 cells per well for the astrocytes and melanocytes, in 150 µL of cell culture medium. The densities were chosen for each cell lines based on their growth rate, in order to achieve comparable densities for all cell lines at the time of treatment. For the direct treatment, the cells were seeded in a 24-well plate with a density of 8333 cells per well for A549, BEAS-2B, and A375; 10,000 cells per well for U87; and 20,000 cells per well for the astrocytes and melanocytes, in 500 µL of cell culture medium.

### 4.2. Plasma Sources

**Figure 5:**
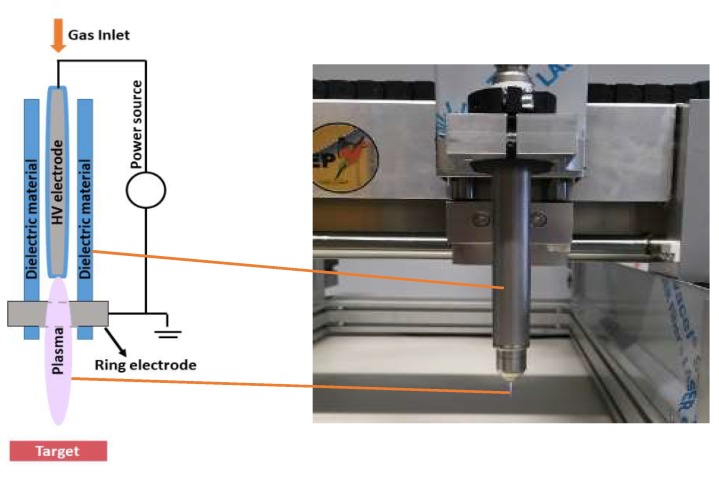
Schematic representation and picture of the kINPen^®^IND used in the experiments.

We studied both direct and indirect plasma treatment. For the indirect treatment, we used the kINPen^®^IND plasma jet (INP Greifswald/Neoplas tools GmbH, Greifswald, Germany). This is an atmospheric pressure argon plasma jet, made of a central pin electrode (1 mm diameter), shielded by a dielectric quartz capillary (internal diameter 1.6 mm and outer diameter 2 mm), which is connected to a grounded ring electrode. The distance from the tip of the central electrode to the exit nozzle is about 3.5 mm (Figure 5). Plasma is generated by applying a sinusoidal voltage to the central electrode with a frequency between 1.0 and 1.1 MHz, and a maximum power of 3.5 W. This voltage creates a gas discharge between both electrodes, which generates the reactive species inside the capillary. These species are carried out with the argon gas flow, creating a plasma effluent with a length of 9–12 mm and a diameter of about 1 mm.

**Figure 6:**
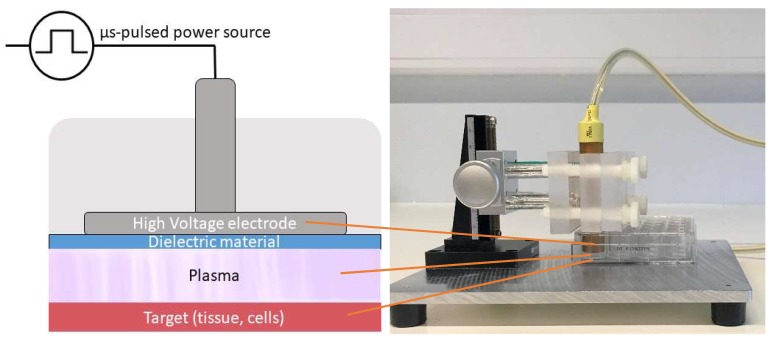
Schematic representation and picture of the FE-DBD used in the experiments.

For the direct plasma treatment, we used a floating-electrode dielectric barrier discharge (FE-DBD) operated at atmospheric pressure in air. A DBD normally consists of a pair of electrodes, of which at least one is shielded with a dielectric material, separated by a small gap filled with a gas [63]. High voltage (HV) is applied to one electrode, while the target, in our case cells in a well on a grounded metal plate, functions as the second electrode. As the discharge takes place in the gap between the HV electrode and the target, no additional carrier gas was used with this plasma source (Figure 6). The plasma was generated by applying microsecond-pulses to the HV electrode from a pulse generator (Advanced Plasma Solutions, Malvern, PA, USA) with an amplitude in the range of 17 kV and a varying frequency between 100 Hz and 500 Hz in our experiments.

### 4.3. Indirect Plasma Treatment

We used the kINPen^®^IND to treat 2 mL phosphate-buffered saline (PBS) (pH 7.3) in a 12-well plate. A gap of 6 mm between the tip of the plasma source and the liquid, a gas flow rate of 1 slm, and a treatment time of 5 min was used. In this case, the gap was small enough to have discharges at the liquid surface, as discharge streamers were visible between the head of the plasma jet and the liquid interface. Here, the liquid surface acts as a third electrode, and the electrons interact with the liquid, resulting in electron impact reactions, which affect the generation of RONS. In our experiments comparing all cell culture media on cancer cells, a gap of 30 mm, a gas flow rate of 3 slm, and a treatment time of 7 min was used. Here, the gap was sufficiently large to avoid the generation of these discharges at the liquid surface. Before treatment of cells with plasma-treated PBS (pPBS), the cells were seeded in a 96 well-plate and incubated for 24 h at 37 °C and 5% CO_2_. For treatment, we applied 30 µL of (diluted) pPBS to each well of the 96-well plate. We also used a control sample, where 30 µL of untreated PBS was added.

### 4.4. Direct Plasma Treatment

After cell seeding into 24-well plates and incubating for 24 h at 37 °C and 5% CO2, the cells were treated with the FE-DBD as stated in our previous work [55]. The cell culture medium was first removed, after which cells were washed with PBS and treated for 10 s at a distance of 1 mm and a frequency varying between 100 Hz and 500 Hz. Immediately after treatment, 500 µL of fresh cell culture medium was added to each well. The control sample was handled in exactly the same way, but without turning on the power source and applying high voltage.

### 4.5. Cell Viability Assay

After treatment, the cells were incubated for 24 h at 37 °C and 5% CO_2_ before the further viability analysis with the sulforhodamine B-method (SRB). The cell culture medium was removed from each well and the cells were first fixed to the plate using 10% trichloro acetic acid (TCA) (Fischer Scientific, A322), 100 µL for a 96-well plate and 400 µL for a 24-well plate. The plates were placed at 4 °C for 1 h, after which the TCA was thoroughly washed away with deionized water. The wells were dried, and a SRB-solution (0.1 w/v % in 1% (v/v) acetic acid, Sigma-Aldrich^®^, Missouri, s1402) was added (100 µL for a 96-well plate and 400 µL for a 24-well plate). After 30 min, the SRB was washed away with 1% acetic acid. The cells were dried again, and tris(hydrocymethyl)aminomethane (TRIS)-buffer (Sigma-Aldrich^®^, 252859) was added to each well (100 µL for a 96 well-plate and 400 µL for a 24-well plate). After 30 min, the absorbance was measured at 540 nm, using a BIO-RAD iMark^TM^ Microplate reader for the 96-well plates and a Tecan Infinite F Plex Microplate reader for the 24-well plates. The cell viability was determined by comparing the absorbance of the treated groups with the untreated control sample.

### 4.6. Analysis of the Influence of the Cell Culture Medium

Since the cancerous cell lines and non-cancerous cell lines have different optimal culture media, it was important to analyze the influence of the media on the plasma treatment results. For this purpose, we tested two of the cancer cell lines—i.e., A549 and A375—with the five different cell culture media (DMEM, RPMI1640, BEGM, AM, and DCBM). We cultured the cells in their recommended medium, but at the moment of seeding, we seeded them in the different media. Both the direct (FE-DBD at 500 Hz) and indirect (pPBS, condition 2) treatment were tested.

### 4.7. Statistical Analysis

All experiments were performed in triplicate, and the results are expressed as the mean with associated standard deviation. Statistical significance was determined using Students *t*-test with Welch’s correction (assuming unequal standard deviation) and displayed on the figure plots as *p < 0.05, **p < 0.01, and ***p < 0.005.

## 5. Conclusions

In this study, we evaluated the influence of the cell type, cancer type, and cell culture medium on the cytotoxic effects of both direct and indirect CAP treatment for cancer. In all cases, we found that the influence of these biological parameters was more pronounced for indirect CAP treatment compared to direct CAP treatment.

When analyzing the influence of the cell type, we found that fibroblasts are more resistant to indirect CAP treatment. Also, the different cancer types gave different responses to CAP treatment, where the lung cancer cell line, A549, was more sensitive, compared to the brain cancer cell line, U87.

For the indirect CAP treatment, we observed a large influence of the cell culture medium on cell cytotoxicity, as the more advanced media virtually negated the effects of treatment. Thus, when comparing the viability of cancerous cells in their standard media with the non-cancerous cells in their advanced media, it is tempting to conclude significant selectivity of treatment for all the cancer types. However, when cytotoxicity was compared for cancerous and non-cancerous cells cultured in the same media, it was obvious that this apparent selectivity was due to the cell culture media and genuine differential sensitivity to indirect CAP. This is an important conclusion, which must be kept in mind to avoid drawing false conclusions in cancer cell selectivity studies.

Taken together, the results of our study demonstrate that biological factors—including cell type, cancer type, and culturing medium—must be taken into account before selectivity of CAP treatment can be claimed. Overlooking these parameters can easily result in misdirected conclusions and false claims of selectivity.

## Supplementary Materials

The following are available online at https://www.mdpi.com/2072-6694/11/9/1287/s1, Figure S1: Comparison of the cell densities of A375 and A549 in the different cell culture media. The results are normalized to the recommended media for the cell line (DMEM for A549 and RPMI1640 for A375) indicated by the blue bars. Data are represented as mean ± standard deviation (SD) of three independent experiments with at least two replicates. Statistical significance of all treatment conditions was compared to untreated. *p < 0.05 (One-way ANOVA). Figure S2: Selectivity analysis for lung cancer using (a) indirect and (b) direct CAP treatment. For the indirect treatment we used different dilutions of plasma-treated PBS (pPBS), using the first condition (5 min treatment with a gas glow rate of 1 slm and a gap of 6 mm) and for the direct treatment we used a 10 second treatment with a gap of 1 mm and two different frequencies of the FE-DBD.
