# Supplementary material for: The Influence of Cell Type and Culture Medium on Determining Cancer Selectivity of Cold Atmospheric Plasma Treatment
Source: Cancers (Basel). 2019 Sep 1;11(9):1287. doi: 10.3390/cancers11091287 (PMC6770138; doi:10.3390/cancers11091287)
Supplement: Supplementary file 1 [file cancers-11-01287-s001.pdf]

cancers

Article

The Influence of Cell Type and Culture Medium on Determining Cancer Selectivity of Cold Atmospheric Plasma Treatment

Eline Biscop, Abraham Lin, Wilma Van Boxem, Jinthe Van Loenhout, Joey De Backer, Christophe Deben, Sylvia Dewilde, Evelien Smits and Annemie Bogaerts

Supplementary Information

1. Influence of the cell culture medium on the cell growth

In order to ensure that the different medium alone did not significantly affect cell growth and death, the cytotoxicity assay was performed on cells 24 hours after incubation and cell density of the cells in the different media was compared to that of their recommended medium: DMEM for A549 and RPMI for A375. While A375 were able to grow in all media, equivalent to that of RPMI, A549 growth was significantly impeded by the BEGM medium ($p<0.05$). Since BEGM appears to have an inherent effect on A549 cells, results on selectivity would be confounded when comparisons are made to cells grown in BEGM (e.g. BEAS-2B). While DCBM media also appeared to have an effect on the A549 lung carcinoma cells, the variation was large and statistical analysis did not show a significant difference. However, this difference was not present in the A375 melanoma cells. This is crucial, as DCBM is used to culture melanocytes, and therefore indicates that comparison of CAP selectivity on melanocytes and A375 melanoma cells could be performed.

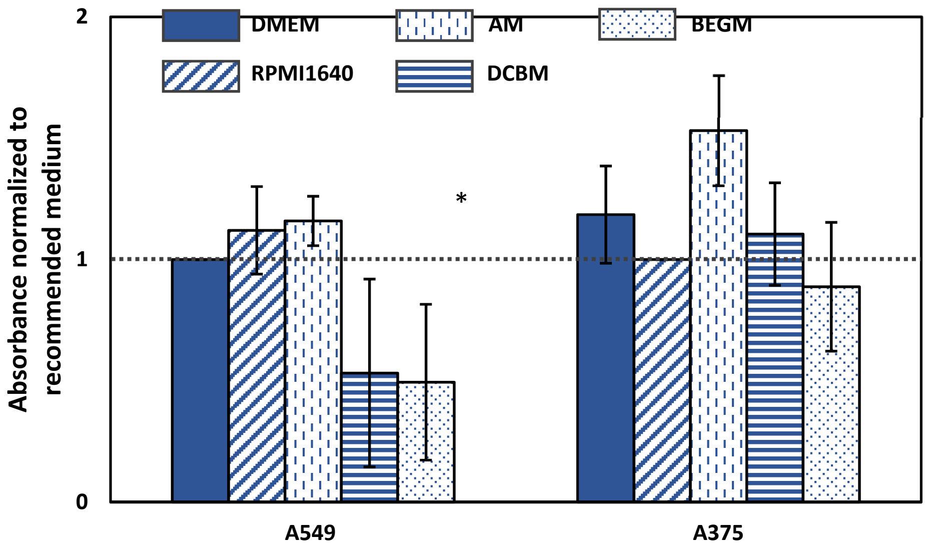

Figure S1. Comparison of the cell densities of A375 and A549 in the different cell culture media. The results are normalized to the recommended media for the cell line (DMEM for A549 and RPMI1640 for A375) indicated by the blue bars. Data are represented as mean ± standard deviation (SD) of three independent experiments with at least two replicates. Statistical significance of all treatment conditions was compared to untreated. *$p<0.05$ (One-way ANOVA).

1. Analysis of the selectivity for A549 and BEAS-2B, for both direct and indirect CAP treatment

A549 in DMEM and BEGM were treated with both direct and indirect CAP and cytotoxicity was compared to that of BEAS-2B treated and cultured in their recommended medium (BEGM). While

under different media conditions, CAP treatment appears to be selective for A549, this selectivity becomes revered when the medium was standardized. This is likely due to the inherent effects BEGM has on A549 cells (Figure S1). Taken together, this further highlights the influence of the cell culture medium on observed biological outcome following CAP treatment.

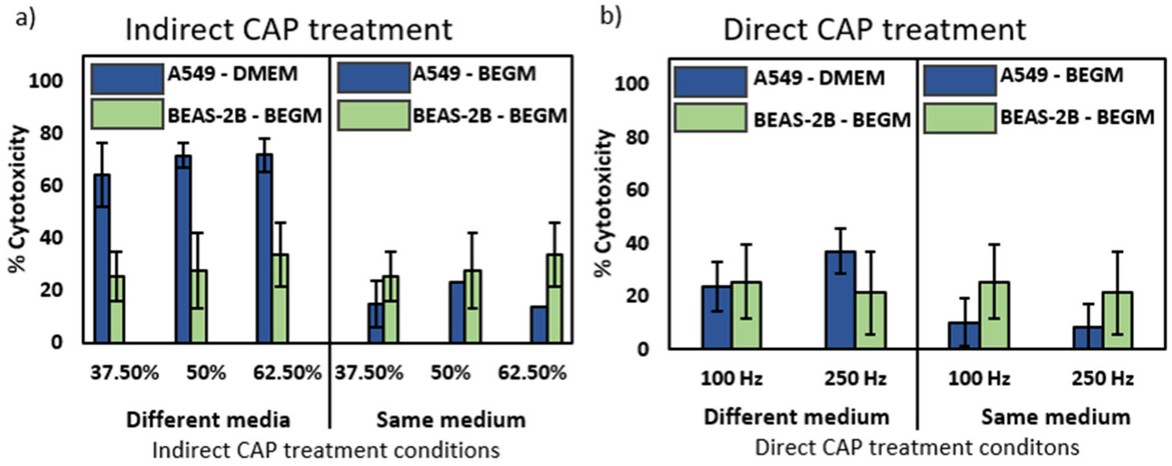

Figure S2. Selectivity analysis for lung cancer using a) indirect and b) direct CAP treatment. For the indirect treatment we used different dilutions of plasma-treated PBS (pPBS), using the first condition (5 minute treatment with a gas glow rate of 1 slm and a gap of 6 mm) and for the direct treatment we used a 10 second treatment with a gap of 1 mm and two different frequencies of the FE-DBD. .
